# Gut microbiota and their metabolites contribute to the heterosis of breast muscle yield in broilers

**DOI:** 10.1186/s40104-026-01409-8

**Published:** 2026-05-11

**Authors:** Qiang Huang, Chaoliang Wen, Shuang Gu, Yuchen Jie, Guangqi Li, Yiyuan Yan, Guiqin Wu, Ning Yang

**Affiliations:** 1 State Key Laboratory of Animal Biotech Breeding and Frontier Science Center for Molecular Design Breeding, China Agricultural University, Beijing, 100193 China; 2 National Engineering Laboratory for Animal Breeding and Key Laboratory of Animal Genetics, Breeding and Reproduction, Ministry of Agriculture and Rural Affairs, Department of Animal Genetics and Breeding, College of Animal Science and Technology, China Agricultural University, Beijing, 100193 China; 3 Sanya Institute of China Agricultural University, Hainan, 572025 China; 4 Beijing Huadu Yukou Poultry Industry Co. Ltd., Beijing, 101206 China

**Keywords:** Breast muscle, Broiler, Cecal microbiota, Heterosis, Metabolites, Nonadditive

## Abstract

**Background:**

Breast muscle yield is a key economic trait in broilers, directly affecting carcass value and profitability, and has been significantly improved by intensive selection and exploiting heterosis through crossbreeding. Our previous work showed that synergy between the gut microbiota and host genome underlies breast muscle heterosis in crossbred progeny (CR) derived from Cornish (CC) and White Plymouth Rock (RR) lines. However, the molecular mechanisms by which the gut microbiota contributes to heterosis in breast muscle yield remain poorly understood. Here, we integrated cecal microbiome, metabolome, and transcriptome data from 266 birds at 42 days of age to elucidate the potential gut microbiota–mediated molecular mechanisms underlying breast muscle yield heterosis.

**Results:**

To assess whether heterosis extends beyond productive traits to the gut microbiota and their metabolites in broilers, we compared the cecal microbial and metabolic profiles of CR with those of their parental lines. The gut microbiota of CR were clearly distinct from those of both parental lines and exhibited heterosis characteristics, with 88 genera displaying heterotic patterns that collectively accounted for approximately 85% of the total microbial abundance. Heterosis was also evident in the cecal metabolites of CR birds. Differential abundance analysis across groups identified 868 cecal metabolites, and abundance-pattern classification showed that approximately 75% exhibited nonadditive patterns in the crossbred progeny. These nonadditive metabolites were predominantly host–microbiota co-metabolites and were mainly enriched in amino acid and lipid metabolic pathways. Importantly, seven of the nine genera previously identified in association with breast muscle yield exhibited heterosis in the crossbred progeny. At the metabolomic level, yield-associated genera were linked to a distinct set of 35 cecal metabolites, dominated by sphingolipids, ether-linked phospholipids, and acyl-homoserine lactones. These metabolites formed coordinated associations with the expression of 269 host genes, which were functionally enriched in MAPK signaling and focal adhesion pathways.

**Conclusions:**

These findings suggest that heterosis exists not only in productive traits but also in gut microbiota and their metabolites, the latter in turn contributed to breast muscle yield, which offers valuable guidance for elucidating the molecular basis of heterosis in animals.

**Supplementary Information:**

The online version contains supplementary material available at 10.1186/s40104-026-01409-8.

## Introduction

Chickens are among the most widely raised animal species globally, providing an essential protein source in the diets of people across diverse countries and regions. Demand for chicken meat has increased substantially because of the growth of the global population and rising living standards, thereby imposing greater demands on the production efficiency of the poultry industry [1]. Broiler selective breeding over recent decades has dramatically increased body weight and breast muscle growth rates [2–4], leading to a substantially shortened production cycle and markedly improved farming efficiency [5]. The breast muscle is an essential component in broilers, and its growth rate and yield are important economic indicators of production efficiency. In modern fast-growing white-feathered broiler breeding programs, increasing breast muscle yield has long been a primary breeding objective, and the breast muscle weight accounts for approximately one-quarter of the total body weight [6, 7]. To further enhance meat production performance, commercial breeding systems typically employ specialized paternal and maternal lines in crossbreeding schemes to utilize heterosis. Heterosis, or hybrid vigor, refers to the biological phenomenon whereby hybrid offspring exceed the parental mean in traits such as growth rate, yield, or adaptability [8]. In crossbreeding experiments between Cornish (CC) and White Plymouth Rock (RR) chickens, the hybrids exhibited positive heterosis for multiple traits, including body weight, average daily gain, breast muscle weight, and leg weight [9, 10]. However, despite the widespread application of crossbreeding in the broiler industry, the molecular regulatory mechanisms through which heterosis contributes to increased major economic traits remain poorly understood and have not been systematically characterized.

According to quantitative genetic theory, heterosis arises from multiple genetic mechanisms, including dominance, overdominance, and epistasis [11]. With the rapid development of sequencing and analysis technologies, integrative analyses based on multi-omics data have been widely applied to dissect heterosis in animals and plants, providing molecular-level insights into the basis of phenotypic heterosis [12, 13]. Crossbreeding between divergent parental lines unites two distinct genomes, creating a novel gene-expression regulatory landscape and reconfiguring molecular regulatory networks, which is widely regarded as a major determinant of heterosis [14]. Recent comparative studies across multiple molecular layers have shown that differentially expressed genes, metabolites, and proteins in crossbred progeny often display nonadditive patterns [15–17]. In chickens, heterosis-related research has primarily focused on traits such as egg production [18], growth and development [9, 19], and fat deposition [20]. These studies have shown that differentially expressed genes in hybrids commonly show nonadditive expression and are enriched in essential biological processes such as cell proliferation and differentiation, energy metabolism, and immune regulation [9].

The gut microbiota is widely regarded as the host’s “second genome”, and growing evidence indicates that it plays a pivotal role in nutrient utilization, energy metabolism, immune homeostasis, and growth performance in poultry [21]. The cecum is a major digestive organ in chickens and the primary site of microbial fermentation in the gastrointestinal tract. In contrast to the rapid peristalsis of the small intestine, digesta is retained longer in the cecum, creating a favorable anaerobic environment for microbial fermentation [1, 22]. Accordingly, the cecum harbors the highest microbial richness and diversity among intestinal segments [23]. Previous studies have shown that cecal microbiota plays an important role in regulating feed efficiency [24], fat deposition [25], and meat quality [26]. The cecal microbiota ferments indigestible carbohydrates under anaerobic conditions to produce short-chain fatty acids (SCFAs), including acetate, propionate, and butyrate [27]. These metabolites can cross the intestinal barrier and enter the circulation, where they regulate host metabolism and gene expression through multiple signaling pathways, functioning as key mediators of gut microbiota–host developmental interactions [28]. Our previous work demonstrated that the gut microbiota, together with host genetics, contributes to the heterosis of breast muscle yield in broilers [9]. However, whether the gut microbiota modulates host gene expression through specific metabolic pathways to regulate breast muscle growth remains largely unresolved.

Given that 42 days of age is the typical market age in commercial broiler production, sampling at this point enables the precise evaluation of key economic traits and the molecular regulatory mechanisms underlying them. In this study, cecal microbiome, transcriptome and metabolome profiles were generated and analyzed for 266 birds from CC and RR purebred lines and their crossbred progeny (CR) at 42 days of age. Building on our previous finding that the gut microbiota and host genome act synergistically to drive heterosis in breast muscle yield, this study further incorporates gut metabolomic data. Through multi-omics data integration, we systematically delineated how the gut microbiota, by influencing intestinal metabolites, modulate host gene expression to promote heterosis in breast muscle yield. This study provides important information on the molecular mechanisms underlying heterosis in broiler chickens and delivers a solid foundation for innovation in broiler breeding and improvement of meat production in the poultry industry.

## Materials and methods

### Experimental animals and sample collection

**Fig. 1:**
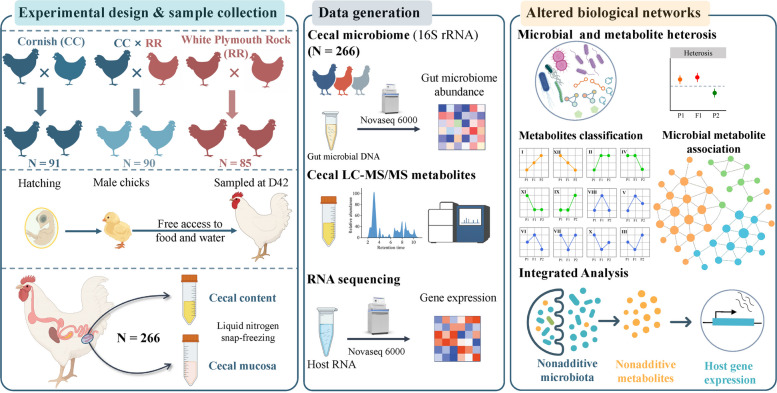
Study design overview. This schematic provides an overview of the establishment of parental and hybrid groups, as well as the sequencing strategies and downstream analysis workflow

An overview of the experimental design and analyses is presented in Fig. 1. All fertilized eggs of CC and RR purebred lines, as well as their crossbred progeny (CC males × RR females), were obtained from Beijing Huadu Yukou Poultry Breeding Co., Ltd. (Beijing, China). Each experimental group consisted of offspring from 20 sire families to ensure genetic diversity, with each rooster mated to six unrelated hens to minimize inbreeding. The eggs were disinfected using 0.1% benzalkonium bromide and then incubated in an incubator set at 37.8 °C and 60% relative humidity for 21 d until hatching. After hatching, males and females were sexed, and 200 healthy male chicks were selected from each group (CC, CR, and RR). These selected chicks were then assigned unique wing band numbers for individual identification. To minimize potential confounding effects, the birds were randomized within cage tiers and housed in group cages under identical environmental conditions with free access to feed and water. The experimental individuals were raised under standardized temperature and illumination conditions throughout the growth period in accordance with the Huadu Yukou broiler management guidelines. At 42 days of age, a total of 266 birds (CC = 91, CR = 90, RR = 85) were retained for subsequent analyses. All birds were humanely euthanized by cervical dislocation. Cecal mucosa and cecal contents were immediately collected, snap-frozen in liquid nitrogen, and stored at −80 °C until further processing.

### Microbial DNA and RNA sequencing

Gut microbial DNA was extracted from cecal contents using the QIAamp DNA Stool Mini Kit (QIAGEN, Hilden, Germany), while host RNA was isolated from cecal mucosa using the Tiangen RNA extraction kit (Tiangen Biotech, Beijing, China). All samples that passed quality control were subjected to sequencing. The detailed procedures for 16S rRNA sequencing and RNA-seq library preparation, as well as sequencing quality control and preprocessing pipelines, have been described in our previous study [9]. Briefly, the V4 region of the bacterial 16S rRNA gene was amplified using universal primers and sequenced on an Illumina platform with paired-end reads. For host transcriptome profiling, RNA libraries were constructed and sequenced using the Illumina NovaSeq 6000 system to generate 150-bp paired-end reads.

### 16S rRNA and transcriptome data preprocessing

Raw microbiome data were processed using the QIIME 2 (ver 2020.8) workflow, including read filtering, chimera removal, amplicon sequence variant (ASV) inference, and taxonomic classification using the SILVA database [29]. The ASV table was imported into the phyloseq package (ver 1.38.0) for downstream analysis [30]. Alpha diversity indices (Simpson index and observed genus richness) were calculated using the vegan package, and group differences were evaluated using pairwise Wilcoxon rank-sum test. Beta diversity was assessed using Bray–Curtis distances and visualized using Non-metric multidimensional scaling (NMDS) ordination. Taxonomic profiles at the phylum level were summarized and visualized as stacked bar plots. RNA-seq reads were trimmed for adapters and low-quality bases, aligned against the chicken reference genome (GRCg7b) using HISAT2 (ver 2.2.1) [31, 32], and gene expression levels were quantified using StringTie (ver 2.1.5) [33]. Based on our previously established dominance classification strategy, 8,684 nonadditive genes were identified and subsequently used in downstream multi-omics analyses [9].

### Microbial heterosis and network analysis

Microbial genera present in more than 60% of samples were retained for downstream analysis, and their relative abundances were calculated. To assess hybrid effects on microbial communities, microbiome heterosis rate (H%) was calculated at the genus level based on the relative abundances of each genus in the two parental lines and the crossbred progeny. The heterosis coefficient was calculated, and statistical significance was assessed following Mai et al. [19]. Genera exhibiting significant deviation from the mid-parent value (*P* < 0.05) were considered to display positive or negative heterosis.

For microbial interaction analysis, co-occurrence networks were constructed for each group using Spearman correlations (|*r*| > 0.4, FDR < 0.05). Network topological properties were calculated with the igraph package (ver 2.2.1) [34], and network visualization was performed using Gephi (ver 0.9.3) [35].

### Cecal metabolite extraction

Cecal metabolites were extracted using methanol–water extraction. Approximately 30 mg of homogenized cecal contents was transferred to a 1.5-mL microcentrifuge tube and mixed with 400 μL of pre-chilled methanol–water (4:1, v/v) containing a mixture of internal standards (4 μg/mL). After incubation at −40 °C for 2 min, the samples were mechanically disrupted (60 Hz, 2 min) and then sonicated in an ice–water bath for 10 min. The mixtures were incubated at −40 °C overnight and centrifuged at 12,000 × *g* for 10 min at 4 °C. Subsequently, 150 μL of the supernatant was filtered through a 0.22-μm membrane filter and transferred to autosampler vials. All extracts were stored at −80 °C until Liquid chromatography–mass spectrometry (LC–MS) analysis. A pooled quality control (QC) sample was prepared by combining equal aliquots from all extracts and was injected at regular intervals throughout the analytical batch to monitor system stability.

### LC–MS/MS data acquisition and preprocessing

Untargeted metabolomic profiling was performed using a Waters ACQUITY UPLC I-Class Plus system coupled to a Q Exactive HF high-resolution mass spectrometer (Thermo Fisher Scientific). Chromatographic separation was achieved on an ACQUITY UPLC HSS T3 column (100 mm × 2.1 mm, 1.8 μm) maintained at 45 °C. The mobile phases consisted of water containing 0.1% formic acid (A) and acetonitrile (B), with a flow rate of 0.35 mL/min and an injection volume of 2 μL. The gradient elution program began at 95% A, followed by a gradual decrease to 0% A, and then a return to the initial conditions.

The mass spectrometer was operated under both positive and negative electrospray ionization modes with a mass scan range of *m/z* 70–1,050. Full MS and data-dependent MS/MS spectra were acquired at resolutions of 60,000 and 15,000, respectively, using stepped normalized collision energies of 10, 20, and 40 eV. Source parameters included a spray voltage of +3.8 kV (positive mode) and −3.0 kV (negative mode), capillary temperature of 320 °C, sheath gas of 35 arb, and auxiliary gas of 8 arb.

Raw LC–MS data were processed using Progenesis QI (v3.0, Nonlinear Dynamics) for peak detection, retention time alignment, normalization, and deconvolution. Metabolite annotation was performed by matching accurate mass, MS/MS fragmentation patterns, isotope distributions, and retention time against entries in public databases (HMDB, METLIN, and LIPID MAPS) and the in-house LuMet-Animal 3.0 library. Compounds were assigned confidence levels based on predefined annotation scoring criteria. Only metabolites classified as Level 1 (RT ± 0.3 min and fragmentation score ≥ 45) or Level 2 (RT ± 0.3 min and fragmentation score < 45) were retained for subsequent differential-abundance analysis. Features with > 50% missing values within a group were removed, and remaining missing values were imputed with half the minimum non-zero intensity across the dataset to generate a quantitative data matrix for downstream analyses.

### Metabolite data processing and heterosis analysis

Processed metabolite data matrices were imported into R for statistical analysis. Prior to downstream analyses, metabolite intensities were log10-transformed and scaled prior to analysis. Multivariate separation among groups was evaluated using partial least squares–discriminant analysis (PLS-DA) implemented in the ropls R package, with model performance evaluated by cross-validation [36]. To evaluate hybrid performance at the metabolic level, the H% was calculated based on log-transformed relative metabolite contents from the two parental lines and the hybrid group, and statistical significance was assessed accordingly.

Metabolites contributing to group separation were identified using a variable importance in projection (VIP) threshold of > 1 and were subsequently tested for differences in abundance among groups. Differential abundant metabolites were classified into additive and nonadditive patterns using the 12 abundance pattern types established in Mai et al. [19] and applied in our previous study [9]. Briefly, these patterns were determined based on differences in metabolite profiles among the crossbred progeny and the two parental lines. Additivity (types I and XII) was defined when the relative metabolite levels significantly differed between the two parental lines (*P* < 0.05), and the levels in the hybrids was intermediate between them. Dominance (types II, IV, IX, and XI) was assigned when hybrid values did not differ significantly from one parental line but were significantly higher or lower than those in the other. Overdominance (types III, V, VI, VII, VIII, and X) occurred when hybrid values were significantly higher or lower than those in both parental lines. Nonadditive metabolites were defined as those deviating from mid-parent expectations and exhibiting dominance or overdominance patterns. Biological origins and functional pathways of nonadditive metabolites were annotated using MetOrigin to distinguish host-derived, microbiota-derived, co-metabolized, and diet/environment-related metabolites and to perform pathway enrichment analysis [37].

### Microbiota–metabolite association analysis

To explore coordinated hybrid effects between the gut microbiota and cecal metabolome, we focused on microbial genera and metabolites that jointly exhibiting nonadditive patterns. Microbial relative abundances were transformed using centered log-ratio (CLR) transformation, and metabolite data were processed as described above. Pairwise Spearman’s rank correlations were calculated between selected microbial taxa and metabolites. Microbial taxa–metabolite pairs with an absolute correlation coefficient (|*r*|) > 0.4 and a false discovery rate (FDR)–adjusted *P* value < 0.05 were considered significant. Additionally, metabolite–metabolite correlations with |*r*| > 0.6 were retained to delineate metabolic co-regulation relationships. Significant associations were visualized as correlation networks using Gephi, and heatmaps were generated to display the corresponding correlation matrices. Metabolites associated with microbial taxa were subjected to Kyoto Encyclopedia of Genes and Genomes (KEGG) pathway enrichment analysis to identify biological processes potentially mediating microbiota-driven host metabolic regulation.

### Multi-omics integration analysis

To elucidate how key gut microbial taxa contribute to breast muscle heterosis, we integrated microbiome, metabolome, and transcriptome profiles. Based on our previous study [9], nine microbial genera were associated with breast muscle traits; among them, seven genera in the present dataset exhibited significant heterosis (including both positive and negative patterns) and were therefore selected for integrative analysis. We focused on the significant microbial taxa–metabolite associations identified in the correlation analysis described above, and visualized the relationships between these seven genera and nonadditive metabolites. Host gene expression data were normalized using the variance-stabilizing transformation in the DESeq2 package (ver 1.34.0) [38], and genes previously classified as nonadditive were extracted. Metabolites associated with focal microbial genera were linked to their correlated nonadditive genes to construct microbial taxa–metabolite–gene regulatory cascades. Representative association networks and heatmaps were generated to illustrate these relationships. Genes associated with metabolites linked to microbial taxa were further subjected to KEGG pathway enrichment analysis using KOBAS 3.0 [39]. Pathways with *P* < 0.05 were considered significantly enriched.

## Results

### Gut microbiota of crossbred broilers exhibits heterosis and distinct microbial composition

**Fig. 2:**
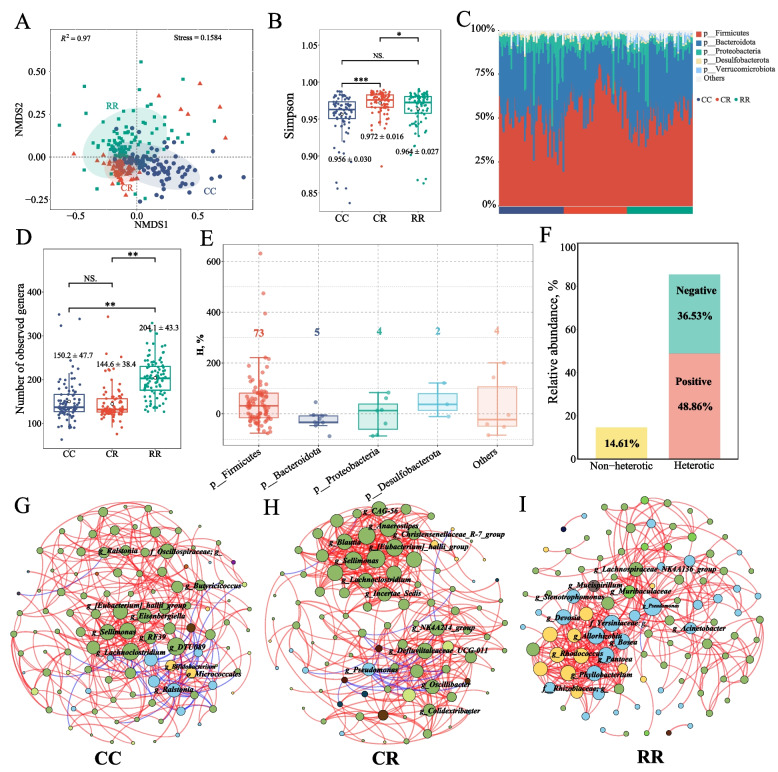
Microbial heterosis and cecal core genera in crossbred progeny and parental lines. **A** NMDS plot based on Bray–Curtis distances showing microbial community structure among Cornish (CC), crossbred progeny (CR), and White Plymouth Rock (RR) chickens. Each point represents a single sample (Stress = 0.158). **B** Simpson α-diversity index of the microbial communities in the three groups. ns, *, and ** indicate *P* > 0.05,* P* < 0.05, and *P* < 0.01, respectively. **C** Relative abundance of major bacterial phyla in the cecal microbiota of CC, CR, and RR chickens. **D** Number of microbial genera detected in each group. **E** Heterosis rate of microbial genera in the CR group. **F** Cumulative relative abundance of genera with positive (red) and negative (blue) heterosis in the CR group. **G–I** Microbial co-occurrence networks for CC (**G**), CR (**H**), and RR (**I**) groups. Nodes are colored by phylum: green, Firmicutes; blue, Proteobacteria; yellow, Actinobacteriota; brown, Bacteroidota

To characterize cecal microbial variation among the three broiler groups, we performed 16S rRNA sequencing and compared community structure, diversity, H%, and microbial association networks. NMDS analysis based on Bray–Curtis distances revealed distinct separation of microbial communities among CC, CR, and RR chickens (Stress = 0.158), indicating that crossbreeding substantially reshaped the gut microbial community structure in the crossbred progeny (Fig. 2A). Microbial α-diversity exhibited a clear line-dependent pattern, with RR exhibiting the highest Simpson index and genus richness, followed by CR and CC (Fig. 2B and D). At the phylum level, Firmicutes and Bacteroidota dominated across all groups, collectively accounting for the majority of sequences; notably, CR displayed a higher abundance of Firmicutes than both CC and RR, suggesting a hybrid-specific compositional shift (Fig. 2C). To evaluate hybrid effects at the microbial taxon level, we calculated heterosis rates for genera detected in at least 60% of samples. In total, 52 genera exhibited positive heterosis, whereas 36 genera showed negative heterosis, representing 85.39% of the total relative abundance in CR (Fig. 2E and F; Additional file 1: Table S1).

Microbial co-occurrence networks revealed distinct genotype-dependent interaction patterns (Fig. 2G–I). In the CC group, the network was characterized by Firmicutes-dominated modules, with key nodes belonging to *Eubacterium hallii group, Lachnoclostridium*, *Sellimonas*, and *Subdoligranulum*. The CR network also showed Firmicutes-centered architecture and shared key hub genera with CC, including *Lachnoclostridium*, *Eubacterium hallii group*, and Sellimonas, while additionally identifying *Oscillibacter* and *Blautia* as dominant nodes. In contrast, the RR network differed markedly from CC and CR, displaying increased contributions from Proteobacteria and Actinobacteriota, with major nodes including *Pseudomonas*, *Pantoea*, *Stenotrophomonas*, and *Alcaligenes*. Overall, the CR microbiome network more closely resembled that of CC than RR, indicating a paternal-line-biased microbial architecture in the hybrid population. Together, these findings indicate that crossbreeding reshapes both the composition and interaction networks of the gut microbiome, establishing a Firmicutes-enriched, high-diversity, and heterosis-associated microbial ecosystem in crossbred broilers.

### Cecal metabolomic profiles of hybrids also display heterotic characteristics

**Fig. 3:**
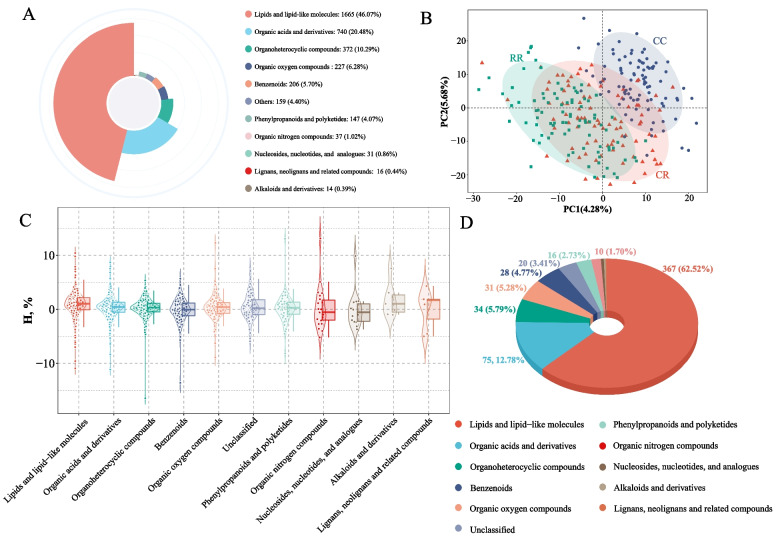
Cecal metabolomic profiling and heterosis analysis in crossbred progeny. **A** Profile of cecal metabolites detected by untargeted LC–MS. **B** PLS-DA score plot of cecal metabolomes from CC, CR, and RR groups. Each point represents a single sample. **C** Distribution of heterosis rates for metabolites with variable importance in projection (VIP) > 1. Each point represents one metabolite, classified according to chemical class. **D** Number of metabolites exhibiting significant heterosis within each chemical class. The number and percentage of significant metabolites are indicated

Untargeted LC–MS-based metabolomic profiling detected a broad spectrum of cecal metabolites spanning diverse chemical classes (Fig. 3A). Lipids and lipid-like molecules constituted the largest category, accounting for 46.07% of all annotated features, followed by organic acids and derivatives (20.48%) and organoheterocyclic compounds (10.29%). Together, these three classes constituted more than 75% of all detected metabolites, indicating that lipid-related and small organic molecules dominate the cecal metabolic landscape in broilers. To examine metabolic divergence among genetic groups, PLS-DA was performed (Fig. 3B). PLS-DA clearly separated CC, CR, and RR individuals along the first two components (Component 1 = 4.28%; Component 2 = 5.68%), with CR samples positioned in an intermediate yet distinct metabolic space relative to both parental lines. This pattern indicates that crossbreeding is associated with substantial remodeling of the cecal metabolome and resulting in a hybrid-specific metabolic profile.

To examine hybrid metabolic performance, metabolites with VIP > 1 were evaluated for heterosis (Fig. 3C). Across metabolic subclasses, the heterosis rate displayed broad distributions, with most compound classes containing metabolites exhibiting both positive and negative deviations from the midparent expectations, indicating complex patterns of metabolic inheritance (Additional file 1: Table S2). Significance testing further demonstrated that heterosis was not limited to any specific biochemical class (Fig. 3D). Lipids and lipid-like molecules accounted for the largest proportion of metabolites exhibiting heterosis (367, 62.52%), followed by organic acids and derivatives (75, 12.78%) and organoheterocyclic compounds (34, 5.79%). Minor contributions were observed from nucleosides, benzenoids, organic oxygen compounds, and other minor classes. These results indicate that crossbreeding is associated with extensive alterations in cecal metabolism, with lipid metabolic pathways showing the strongest response.

### Nonadditive cecal metabolites arise from host–microbiota co-metabolism

**Fig. 4:**
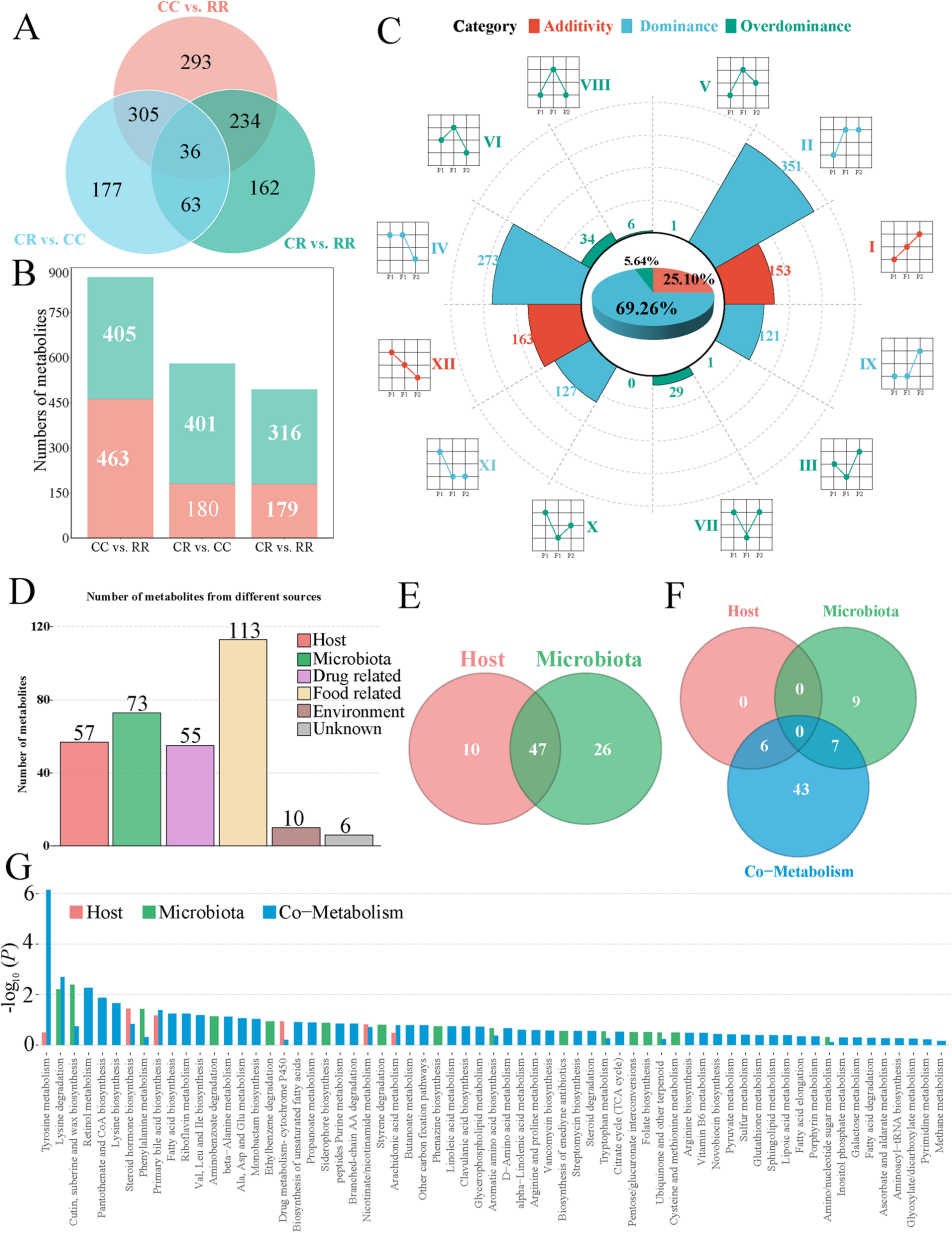
Analysis of metabolite patterns and functional enrichment of nonadditive metabolites in crossbred progeny. **A** and **B** Overall differential metabolites. **A** Venn diagram depicting the number of unique and common differential metabolites among pairwise comparisons of the three broiler groups. **B** Histogram displaying the overall count of upregulated and downregulated differential metabolites in each comparison.** C** Abundance patterns for differential metabolites. The pie chart shows the percentage distribution of additivity, dominance, and overdominance patterns for metabolites in the CR group. The stacked bar chart presents the counts of metabolites classified into four categories: abundance level up, abundance level down, transgressive high-abundance, and transgressive low-abundance. **D **and** E** Identification of nonadditive metabolites from various sources. **D** Number of annotated nonadditive metabolites derived from the host, microbiota, or through co-metabolism. **E** Venn diagram showing the overlap of nonadditive metabolites between host-derived and microbiota-derived categories. **F** and **G** Metabolic pathway analysis of nonadditive metabolites. **F** The number of enriched metabolic pathways derived from host, microbiota, or co-metabolism sources. **G** Metabolic pathway enrichment analysis for nonadditive metabolites belonging to the different sources, displayed as a bar chart. The color of bars indicates the source of the metabolites

We investigated differences in relative metabolite contents between two purebred lines and hybrids. In pairwise comparisons, 868 metabolites were differentially abundant in CC vs. RR (405 up, 463 down), 581 in CR vs. CC (401 up, 180 down), and 495 in CR vs. RR (316 up, 179 down) (Fig. 4A and B). We then classified all differential metabolites according to their difference patterns relative to the two parental lines (Fig. 4C). More than 95% of metabolites were successfully assigned; additivity, dominance, and overdominance accounted for 25.10%, 69.26%, and 5.64%, respectively, indicating that nonadditive categories predominate in the crossbred progeny. The number of abundance level up metabolites, abundance level down metabolites, transgressive high-abundance metabolites, and transgressive low-abundance metabolites in the crossbred progeny were 624, 248, 69, and 2, respectively.

Among the nonadditive metabolites with source annotations, 57 were classified as host-derived and 73 as microbiota-derived, with 47 metabolites shared between the two categories (Fig. 4D and E). To further explore the biological functions associated with nonadditive metabolites, we employed MetOrigin to annotate and analyze their functional pathways. As shown in Fig. 4F, co-metabolism-related pathways accounted for the largest proportion (43 pathways), followed by microbiota-derived pathways (9 pathways), whereas no host-exclusive pathways were detected. In addition, 6 pathways were shared between host and co-metabolism, and 7 pathways were shared between microbiota and co-metabolism, while no pathway was jointly assigned to all three categories, indicating that host–microbiota co-metabolic processes represent the predominant functional source of nonadditive metabolites. We next examined the enriched pathways associated with these metabolites (Fig. 4G). Strikingly, co-metabolic pathways dominated the enrichment landscape, particularly those related to tyrosine metabolism, lysine degradation, and cutin, suberine and wax biosynthesis, which showed the strongest enrichment significance. Several key amino-acid metabolism pathways were also prominently enriched, including valine, leucine and isoleucine biosynthesis, phenylalanine metabolism, and glutamate and aspartate metabolism, suggesting an important role of amino-acid utilization in crossbred metabolic regulation. Notably, lipid-associated pathways such as retinol metabolism, fatty acid biosynthesis, and linoleic acid metabolism were also significantly enriched. Together, these findings indicate that nonadditive metabolites in crossbred broilers are primarily associated with amino-acid and lipid metabolic pathways, driven largely through host–microbiota co-metabolic interactions.

### Microbiota–metabolite association and potential functions

**Fig. 5:**
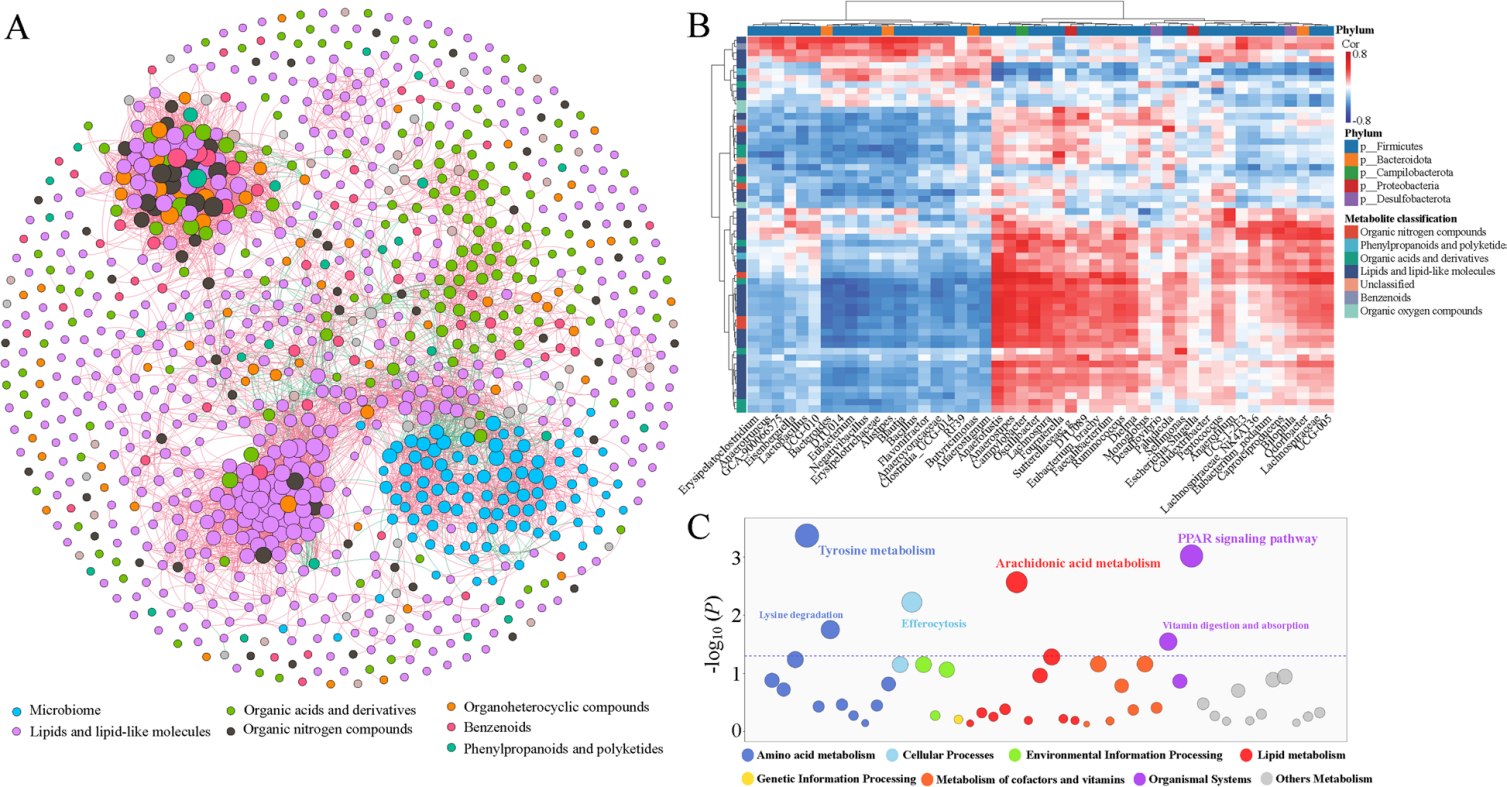
Microbiota–metabolite association and functional enrichment analysis. **A** Correlation network between nonadditive microorganisms and metabolites. Nodes represent microbial genera or metabolites, with node size proportional to the number of connections. Edges represent significant Spearman correlations between nodes (|*r*| > 0.4, FDR < 0.05), with red and blue indicating positive and negative correlations, respectively. **B** Heatmap of Spearman correlation coefficients between significantly associated microbial genera and metabolites. Rows and columns represent metabolites and microbial genera, respectively, and color intensity reflects correlation strength. **C** Functional enrichment analysis of metabolites significantly correlated with gut microbiota. Bubble size represents −log_10_(*P*). The purple dashed line indicates the threshold for significance (*P* = 0.05)

To elucidate the interactions between nonadditive microorganisms and metabolites, a correlation network was constructed using significant Spearman correlations (|*r*| > 0.4, FDR < 0.05). As shown in Fig. 5A, the resulting network exhibited a highly interconnected structure composed of multiple distinct clusters. Microbial nodes were predominantly associated with metabolites classified as lipids and lipid-like molecules, organic acids and derivatives, and organic nitrogen compounds, indicating extensive co-regulatory relationships between microbial genera and host- or diet-derived metabolites. To further characterize these associations, a heatmap was generated to visualize pairwise correlations (Fig. 5B). Detailed correlation coefficients are provided in Additional file 1: Table S3. Functional enrichment of significantly correlated metabolite–microbial taxa pairs (Fig. 5C) revealed that the associated metabolites were mainly involved in tyrosine metabolism, lysine degradation, and efferocytosis, as well as arachidonic acid and PPAR signaling pathways. Among them, tyrosine metabolism and lysine degradation are part of amino-acid metabolic processes that are essential for energy supply and tissue development, whereas arachidonic acid metabolism and PPAR signaling participate in lipid metabolism and inflammatory regulation. Collectively, these findings suggest that the gut microbiota modulates host amino-acid and lipid metabolic pathways, thereby contributing to physiological and developmental differences in crossbred progeny.

### Nonadditive microbiota-derived metabolites modulate host gene expression

**Fig. 6:**
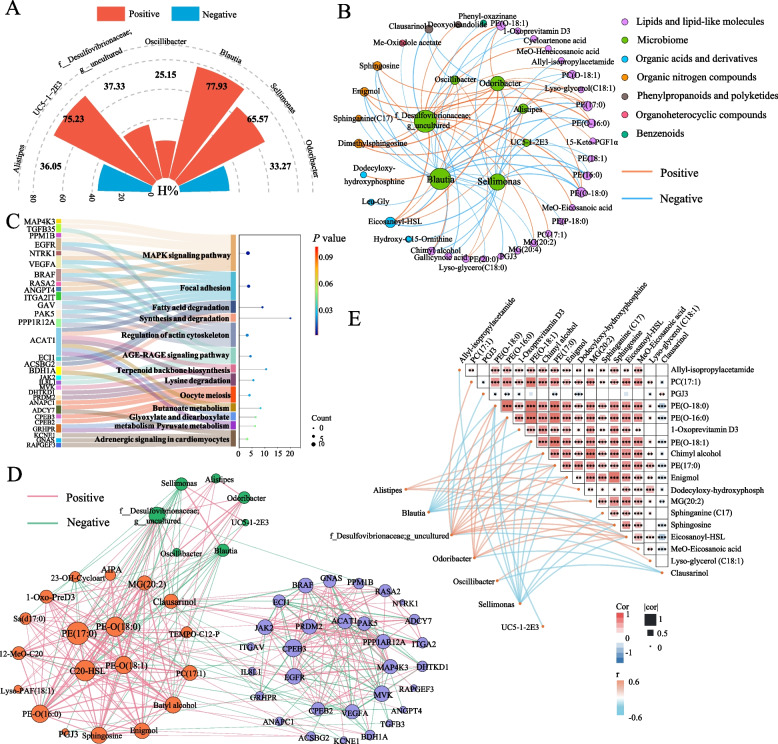
Integrative analysis of breast muscle yield–associated gut microorganisms, metabolites, and host genes. **A** Heterosis rates (H%) of seven breast muscle yield–associated microorganisms in the CR group. The red and blue sectors indicate positive and negative heterosis, respectively. **B** Correlation network between seven microorganisms with significant heterosis and their associated gut metabolites. Edge width represents the absolute strength of the correlation, and edge color denotes positive (red) or negative (blue) associations. **C** Functional enrichment of genes associated with metabolites correlated with the seven microorganisms. **D** Cross-domain association network linking breast muscle yield–associated gut bacteria, metabolites, and host genes. Edge color indicates positive or negative correlations. **E** Heatmap of pairwise correlations between the seven gut microorganisms and their associated metabolites. Asterisks indicate significance levels: ^*^P < 0.05; ^**^P < 0.01; ^***^P < 0.001

Based on our previous findings identifying nine microorganisms significantly associated with breast muscle yield, we further evaluated their heterosis rates in the crossbred progeny. Seven microorganisms showed significant heterosis, with rates ranging from 25.15% to 77.93% (Fig. 6A). Subsequent correlation analysis demonstrated that these yield-associated microorganisms were linked to a defined subset of gut metabolites, comprising 35 cecal metabolites, the majority of which were lipid-related, particularly sphingolipids, ether-linked phospholipids, and acyl-homoserine lactones (Fig. 6B). *Blautia*, *Odoribacter*, and *f._Desulfovibrionaceae;g._uncultured* showed predominantly positive correlations with lipid and nitrogen compounds metabolites, whereas *Sellimonas* exhibited mainly negative associations with these metabolites, suggesting divergent metabolic regulation among key genera. Further integration of metabolomic and transcriptomic data revealed that microorganism-associated metabolites were significantly linked to the expression of 269 host genes (Additional file 1: Table S4). Functional enrichment analysis further revealed that 30 of these genes contributed to significantly overrepresented pathways, including MAPK signaling, focal adhesion, regulation of the actin cytoskeleton, and AGE–RAGE signaling, as well as fatty acid and lysine degradation (Fig. 6C). The integrative correlation network further showed that these microorganisms could potentially influence host gene expression by modulating intestinal metabolites, thereby participating in the molecular regulation of breast muscle yield (Fig. 6D). The overall association landscape between the seven microorganisms and their related gut metabolites was depicted in Fig. 6E.

## Discussion

Breast muscle yield directly affects production performance and profitability [7]. Elucidating the genetic basis of heterosis for this trait is essential for understanding yield formation and for guiding breeding strategies to sustain global poultry meat production. Our previous work showed that crossing Cornish with White Plymouth Rock increases growth performance and breast muscle yield in the crossbred progeny, with evidence that heterosis arises from coordinated interactions between the gut microbiota and the host genome [9]. However, the metabolic pathways and signaling pathways through which the microbiota shape host phenotypes remain unclear. Building on these findings, we integrated metabolomics with 16S rRNA and transcriptome data to dissect the microbiota–metabolite–host gene axis. This multi-omics framework delineates how the gut microbiota contribute to breast muscle yield heterosis in broilers, revealing crossbreeding-driven remodeling of the intestinal ecosystem and its metabolic networks, and providing mechanistic insight into microbiota-mediated heterosis in growth traits.

The gut microbiota plays a pivotal role in regulating nutrient absorption, metabolic stability, and growth performance in poultry. Growing evidence indicates that the intestinal microbial community actively participates in shaping key economic traits in chickens, including feed efficiency, abdominal fat deposition, and breast muscle yield [9, 24, 40]. The shift toward a higher relative abundance of Firmicutes and a lower abundance of Bacteroidetes in hybrids may reflect enhanced microbial capacity for energy harvest. Previous studies have shown that an increased Firmicutes-to-Bacteroidetes ratio is associated with elevated production of butyrate and other short-chain fatty acids, improved intestinal energy extraction, and greater lipid deposition [41, 42]. Network analysis showed that the hybrids’ microbial co-occurrence network had higher connectivity and stronger modularity, with hub genera mainly from *Lachnoclostridium*, the *Eubacterium hallii group*, and *Sellimonas*. These genera produce SCFAs (e.g., butyrate, propionate), which fuel intestinal epithelial cells and enhance barrier integrity by upregulating tight-junction proteins [41, 43]. By contrast, the RR group had a higher relative abundance of Proteobacteria, a phylum often linked to intestinal inflammation and reduced metabolic efficiency [44]. Collectively, these findings suggest that hybridization may enhance cooperative interactions among metabolically beneficial genera while reducing the influence of potential pathogens, thereby improving host production performance.

The intestinal metabolome reflects the integrated metabolic output of the host and its resident microbiota, serving as a composite indicator of energy utilization, nutrient absorption, and metabolic homeostasis [45]. Notably, a substantial proportion of the metabolites exhibiting heterosis were classified as lipids and lipid-like molecules, indicating that lipid metabolism may contribute to the growth advantage observed in hybrids. Lipid-related metabolites are fundamental components of biological membranes and play critical roles in metabolic regulation and signal transduction, thereby potentially supporting enhanced growth performance in hybrid individuals [46]. Beyond functioning as energy stores and core membrane constituents, lipids act as key regulators of systemic metabolic homeostasis [46]. Lipid species are derived from the diet, endogenous host metabolism, and microbial biosynthesis and modification by intestinal commensals [47]. The gut microbiota plays a crucial role in regulating host lipid metabolism [48]. Accordingly, lipid metabolic changes in the hybrids may reflect increased microbial activity that contributes to growth trait heterosis. Thus, elevated lipid metabolites in hybrids likely result from enhanced microbial metabolism, which in turn supports heterosis-related growth performance. Moreover, altered lipid metabolism may influence intestinal immune homeostasis and antioxidant capacity [49]. Together, these multilayered regulatory processes promote metabolic adaptation and improve nutrient utilization efficiency in hybrid progeny.

Most differentially abundant metabolites in the hybrids exhibited nonadditive patterns, predominantly dominance effects. This result is consistent with our previous findings on gene expression and gut microbial composition, indicating that nonadditive regulation is widespread in hybrids [10]. Similar patterns have been reported in hybrid studies of other species, including rice for yield-related traits [50], abalone for thermal tolerance [51], and yellow catfish for traits related to growth, metabolism, and disease resistance [16]. In particular, Mai et al. [19] reported that dominance expression was the predominant gene expression pattern in the breast muscle of crossbred chickens, and this pattern was closely associated with heterosis for growth and meat production traits, particularly body weight and breast muscle yield. Collectively, these findings suggest that dominance effects may represent a major genetic basis underlying heterosis in the crossbred progeny. Functionally, these gut nonadditive metabolites were enriched in energy and amino-acid metabolic pathways, including fatty acid degradation, lysine degradation, tyrosine metabolism, and glutamate/aspartate metabolism. These pathways are critical for regulating energy metabolism, nutrient absorption, and shaping gut microbial community structure [52]. Specifically, amino acids are fundamental building blocks of proteins and metabolic precursors for anabolism, and also participate in nucleotide and lipid biosynthesis [53]. Fatty acid metabolites directly participate in mitochondrial β-oxidation, supplying energy for growth and metabolism [54]. Furthermore, the enrichment of host–microbiota co-metabolic pathways suggests that nonadditive metabolites arise from enhanced metabolic synergy between the host and gut microbiota, thereby improving nutrient conversion efficiency. Collectively, these functional attributes indicate that hybrid offspring adopt a more efficient metabolic state, providing a molecular basis for the heterotic gains in growth and production.

Previous studies have shown that the gut microbiota engages in intimate metabolic crosstalk with the host via microbially derived metabolites, and integrative microbiome–metabolome analyses are among the most effective approaches for exploring host–microbiota interactions [45, 55]. In this study, the nonadditive gut microbial genera in the hybrids were strongly correlated with multiple metabolites, forming a complex, highly interconnected association network. Functional enrichment indicated that metabolites significantly associated with these genera were primarily involved in amino acid metabolism, PPAR signaling, and efferocytosis. These findings suggest that the gut microbiota may regulate nitrogen utilization and energy supply by modulating host amino acid synthesis and degradation. Prior evidence indicates that gut microorganisms can alter amino acid availability and metabolic routing through fermentation, thereby affecting host amino acid homeostasis and the efficiency of protein synthesis [56]. In addition, the gut microbiota not only transforms and synthesizes lipids but also catabolizes luminal lipids into secondary metabolites with regulatory effects on the host [57]. The PPAR signaling pathway is essential for lipid metabolism, energy homeostasis, and immune regulation [58, 59], whereas efferocytosis is essential for maintaining intestinal immune balance and tissue homeostasis [60]. Collectively, these results indicate that the gut microbiota exerts critical metabolic regulatory effects in hybrid offspring by modulating relative metabolite levels and function, thereby influencing host development.

In our previous work, several gut microbial genera were associated with breast muscle yield, yet their specific regulatory pathways remained undefined. Building on these findings, the present study integrates metabolomic data to explore how these microbial taxa may indirectly influence host gene expression via intestinal metabolites. Correlation analysis showed that heterosis-associated microbial taxa were closely linked to multiple lipid metabolites, including sphingolipids and phosphatidylethanolamines. Host genes correlated with these metabolites were mainly associated with key developmental pathways, such as the MAPK signaling pathway, focal adhesion, and regulation of the actin cytoskeleton. These signaling pathways play pivotal roles in muscle cell proliferation, differentiation, and functional maintenance. The MAPK pathway is a well-characterized pathway that orchestrates a broad spectrum of cellular processes, including metabolism, proliferation, and apoptosis [61]. It promotes skeletal muscle development by activating satellite cells and regulating cell cycle progression [62, 63]. Moreover, MAPK signaling is critical for the regulation of skeletal muscle growth and development in broiler chickens [64]. Studies in chickens have shown that alterations in MAPK gene expression affect the proliferative capacity of muscle stem cells [65], and contribute to the regulation of intestinal phosphorus and calcium absorption [66]. Additionally, focal adhesion contributes to sarcomere assembly, muscle hypertrophy, and glucose metabolism, thereby playing an important role in maintaining muscle cell integrity and function [67, 68]. The focal adhesion and actin cytoskeleton regulation pathways jointly mediate mechanotransduction and structural remodeling in muscle cells, providing an essential basis for stable muscle tissue growth [69]. Therefore, the enrichment of these pathways observed suggests that these metabolites may promote breast muscle development by influencing host gene expression. Taken together, these findings indicate that the gut microbiota reshapes the intestinal metabolite landscape and modulates associated signaling and gene regulatory networks, thereby establishing a host–microbiota signaling axis mediated by metabolites that favors muscle development. This provides new molecular evidence for understanding the mechanisms underlying heterosis in breast muscle yield in hybrids. These findings collectively suggest that heterosis in breast muscle yield may be partly driven by coordinated interactions among the gut microbiota, intestinal metabolites, and host gene regulatory networks.

## Conclusion

This study presents a comprehensive multi-omics analysis of the molecular mechanisms underlying breast muscle heterosis in broilers. Crossbreeding was found to reshape both the gut microbiome and metabolome, with the majority of differentially abundant microbiota and metabolites exhibiting heterosis-like features and nonadditive patterns. Moreover, these nonadditive metabolites were predominantly involved in host–microbiota co-metabolic pathways, including tyrosine metabolism, lysine degradation, and fatty acid–related processes, reflecting a remodeled intestinal metabolic landscape in crossbred progeny. Integrative analysis demonstrated that breast muscle yield–associated microbial taxa modulate gut metabolites, which are further connected to nonadditive genes involved in MAPK signaling, focal adhesion, and actin cytoskeleton regulation, thereby shaping muscle growth and structural remodeling. Our findings suggest that crossbred broilers exhibit heterosis in their gut microbiota and metabolites, which in turn contribute to breast muscle growth and development, highlighting the potential for microbiota-associated heterosis in future broiler breeding.

## Supplementary Information

Additional file 1: Table S1. Heterosis rates of microbial taxa in crossbred progeny. Table S2. Heterosis rate of metabolites in crossbred progeny. Table S3. Spearman correlation coefficients between nonadditive microorganisms and metabolites. Table S4. Significant correlations between nonadditive metabolites and associated genes in crossbred progeny.

## Data Availability

The 16S rRNA gene sequencing data from the 266 cecal content samples have been deposited in the Genome Sequence Archive at the China National Center for Bioinformation under accession number CRA016659. The RNA-seq raw data of the cecal mucosa are available under accession number CRA016683.

## Abbreviations

AGE–RAGE: Advanced glycation end products–receptor for AGE
ASV: Amplicon sequence variant
CC: Cornish
CLR: Centered log-ratio transformation
CR: Crossbred progeny (CC × RR)
FDR: False discovery rate
H%: Heterosis rate
LC–MS: Liquid chromatography–mass spectrometry
MAPK: Mitogen-activated protein kinase
NMDS: Non-metric multidimensional scaling
PLS-DA: Partial least-squares discriminant analysis
PPAR: Peroxisome proliferator-activated receptor
RNA-seq: High-throughput paired-end RNA sequencing
RR: White Plymouth Rock
SCFA: Short-chain fatty acid
VIP: Variable importance in projection
